# The complete mitochondrial genome of *Amolops hainanensis* (anura: ranidae)

**DOI:** 10.1080/23802359.2025.2475826

**Published:** 2025-03-07

**Authors:** Chuhan Zi, Zhengyan Zhou, Lanying Xu, Sufan Yu, Lin Ding, Ziyi Liu, Longming Fu, Feng Lin, Xiuzhong Li, Yu Zhou

**Affiliations:** a College of Life Science and Bioengineering, Shenyang University, Shenyang, China; b School of Chemical Safety, North China Institute of Science and Technology, Hebei, China; c College of Life Science, Shenyang Normal University, Shenyang, China

**Keywords:** Torrent frog, illumina sequencing, mitogenome, phylogenetic analysis

## Abstract

The complete mitochondrial genome sequence of *Amolops hainanensis* is 17778 bp in length (GenBank accession number PP734094) and contains 13 protein-coding genes, 2 rRNA genes, and 22 tRNA genes. The mitochondrial gene arrangement of *A. hainanensis* is identical to those of other *Amolops* species and has a typical neobatrachian arrangement. Mitogenomic phylogenetic analysis revealed that *Amolops* is composed of two main clades. *A. hainanensis* is a member of the western clade and split from the basalmost node of the subclade composed of the other species of the western clade of *Amolops*.

## Introduction

*Amolops hainanensis* (Boulenger, 1899), which is commonly referred to as the Hainan Torrent Frog, is a torrent frog that is endemic to China. This frog lives on the rocks of fast-flowing streams or the rock face behind the waterfall at an altitude of 80–850 m (Fei 2020). *A. hainanensis* is distributed only on southern Hainan Island and has a small population size (Fei 2020). The narrow distribution implies that *A. hainanensis* needs more attention in conservation. *A. hainanensis* is listed as an endangered species on China’s Red List of Biodiversity: Vertebrates released by the Ministry of Ecology and Environment and the Chinese Academy of Sciences and level-two protection species to the list of state-protected wildlife coreleased by the National Forestry and Grassland Administration and the Ministry of Agriculture and Rural Affairs.

Vertebrate mitochondrial genomes are circular, typically 14–20 kbp, and contain genes for 13 proteins, 2 ribosomal RNAs, 22 transfers RNAs (Boore 1999; Pereira 2000). Vertebrate mitochondrial DNA is histone free, has limited repair ability, and therefore has a relatively higher mutation fixation rate than the nuclear genome does (Pesole et al. 1999; Jansen 2000). Although vertebrate mitochondria typically have a standard gene arrangement, some species or taxa have evolved distinct gene orders (Montaña-Lozano et al. 2022). Within amphibians, the neobatrachian and vertebrate consensus mt gene orders differ in the relative position of the *trnL*, *trnT* and *trnP* genes, which in neobatrachian mt genomes, are found next to the *trnF* gene downstream of the control region (Irisarri et al. 2012).

In the past few decades, mitochondrial DNA has been used to construct many large-scale phylogenetic frameworks of animals, such as those of Amphibia (Pyron and Wiens 2011), Squamata (Pyron et al. 2013) and Feliformia (Zhou et al. 2017). Currently, mitochondrial genomes or mitochondrial DNA are still the most widely used genomic markers for amphibian population genetics, taxonomy and phylogenetics (such as Zhou et al. 2021; Zhou et al. 2024; Wu et al. 2020). However, the mitochondrial genome sequence of *A. hainanensis* is still lacking. Therefore, for the first time, we sequenced and analyzed the complete mitochondrial genome of *A. hainanensis*. These results provide molecular data for future studies of this species.

## Materials and methods

**Figure 1.**
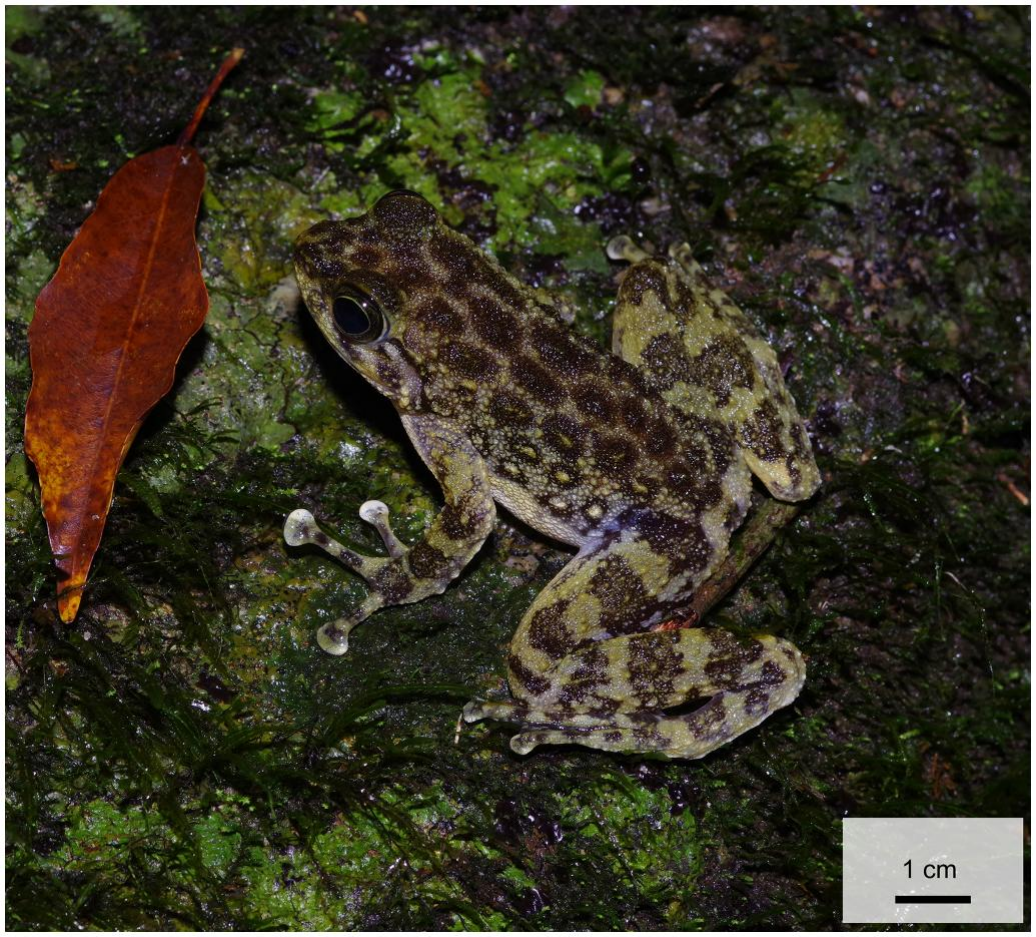
Reference images of *Amolops hainanensis* (Boulenger, 1899). The image was taken by author zhengyan Zhou in Xinglong Tropical Garden, Wanning city, Hainan Province, China. The stored toe-tip tissue (voucher no. ZY-24042102) was taken from the sample in this picture.

One adult *A. hainanensis* was collected from Xinglong Tropical Garden, Wanning city, Hainan Province, China (18.80°N, 110.14°E), on 9 November 2023 (Figure 1). The specimen was morphologically identified as having approximately the same length between the head length and head width, with a round high snout and rather small tympanum, and the dorsal surface was olive or dark brown with irregular black or olive specks and covered with granules and tubercles (Fei 2020). For amphibians, toe-clipping is a commonly nondestructive method used for tissue sampling (Gonser and Collura 1996; Funk et al. 2005; Perry et al. 2011; Ryberg et al. 2013; Holmes et al. 2016, 2023). We collected only a 0.3 cm piece of toe-tip tissue (stored in 95% ethanol) from *A. hainanensis* for genome extraction, after which it was released immediately after the wound was treated with antiseptic agents. Total genomic DNA was isolated from approximately 2 mm^3^ of toe-tip tissue *via* the TIANamp Genomic DNA Kit (TIANGEN Biotech) according to the manufacturer’s instructions. The remainder of the tissue was deposited at Shenyang Normal University, Shenyang, China (Yu Zhou is the contact person: zhouyu1988@outlook.com) under voucher number ZY-23042301. The sequencing library was prepared by Sangon Biotech, Shanghai, China, and sequenced on the Illumina HiSeq 2500 platform with the strategy of 150 paired-end reads. Finally, more than 65 million paired-end reads were sequenced, with an average insert size of 280 bp. The mitochondrial genome was assembled *de novo via* NOVOPlasty v4.3.1 (Dierckxsens et al. 2017) with a partial mitochondrial *COXI* gene sequence of *A. hainanensi* (GenBank: MN961388) (Wu et al. 2020) used as the seed sequence. The average depth of coverage was recovered by using the python script DrawSequencingDepth v1 (Ni et al. 2023). The identical reads from the raw paired-end reads were aligned to the newly assembled *A. hainanensis* mitogenome by using TopHat2 (Kim et al. 2013) under the setting ‘–read-mismatches 0 –read-gap-length 0 –read-edit-dist 0.’ The mitochondrial genome was annotated *via* MITOS2 (Bernt et al. 2013). The mitochondrial genome map of *A. hainanensis* was constructed *via* Chloroplot (Zheng et al. 2020).

**Figure 2.**
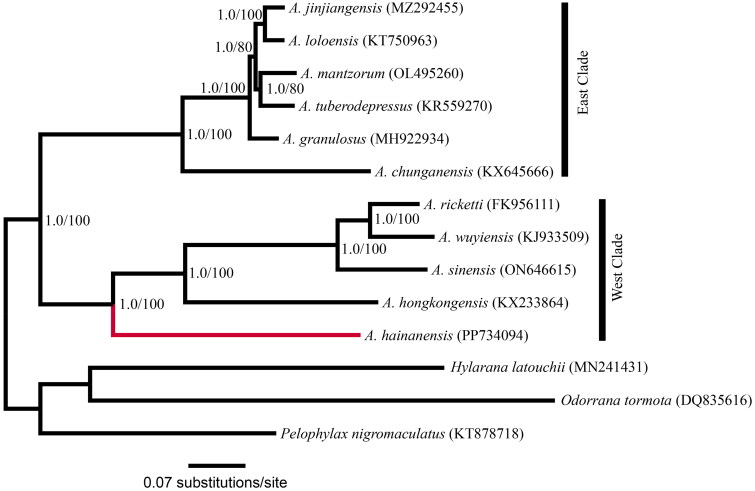
Recovered BI phylogenetic tree of *amolops* based on mitogenomes. The numbers shown between branches indicate the PP (left) and BP (right). The following sequences were used: KJ933509 (Huang et al. 2016), KF956111 (Li et al. 2014), KX233864 (Zhang et al. 2018), KR559270 (Zhang et al. 2016), OL495260 (Shan et al. 2016), MZ292455 (Wang et al. 2021), KT750963 (Xue et al. 2016), MH922934 (Huang et al. 2019), KX645666 (Yuan et al. 2016), ON646615 (Wang and Cao 2021), DQ835616 (Su et al. 2007), MN241431 (Xiao et al. 2019), and KT878718 (Jiang et al. 2017).

The phylogenetic tree was constructed on the basis of 13 protein-coding genes (PCGs). Ten mitogenomes of *Amolops* species available in GenBank were downloaded as ingroup species used for phylogenetic analysis (Figure 2). For each of the 13 PCGs, the DNA sequences were aligned *via* MUSCLE v3.8.31 (Edgar 2004) with the default parameter set. The alignments were further refined *via* Gblocks 9.1b (Castresana 2000) with the ‘codon’ model and other default settings. All refined alignments were then concatenated into the final data set. For the concatenated data set, we manually defined three partitioning strategies: unpartitioned, three partitions (one partition for each codon position), and 13 partitions (one partition for each PCG). Comparisons of the three partitioning strategies and selection of corresponding nucleotide substitution models were conducted with the Bayesian information criterion implemented in PartitionFinder (Lanfear et al. 2012). The 3-partition scheme was chosen as the best-fitting partitioning strategy, and all three partitions favored the GTR + G + I model. The phylogenetic relationships within *Amolops* species were reconstructed *via* both maximum likelihood (ML) and Bayesian inference (BI) methods. Three mitogenomes from the closely related genera *Odorrana, Hylarana* and *Pelophylax* of Ranidae were included as outgroups (Figure 2). Partitioned ML analyses were implemented *via* RAxML version 8 (Stamatakis 2014), with the GTR+G + I model assigned to each partition. Supports for nodes were assessed with 100 rapid bootstrapping replicates. The partitioned BI was implemented in MrBayes 3.2 (Ronquist et al. 2012). We used the Markov chain Monte Carlo (MCMC) approach in BI, running 10,000,000 generations, drawing one sample every 1000, and running four chains per analysis. The effective sample sizes (ESSs) were > 200 for all parameters after the first 10% of generations were discarded.

## Results

**Figure 3.**
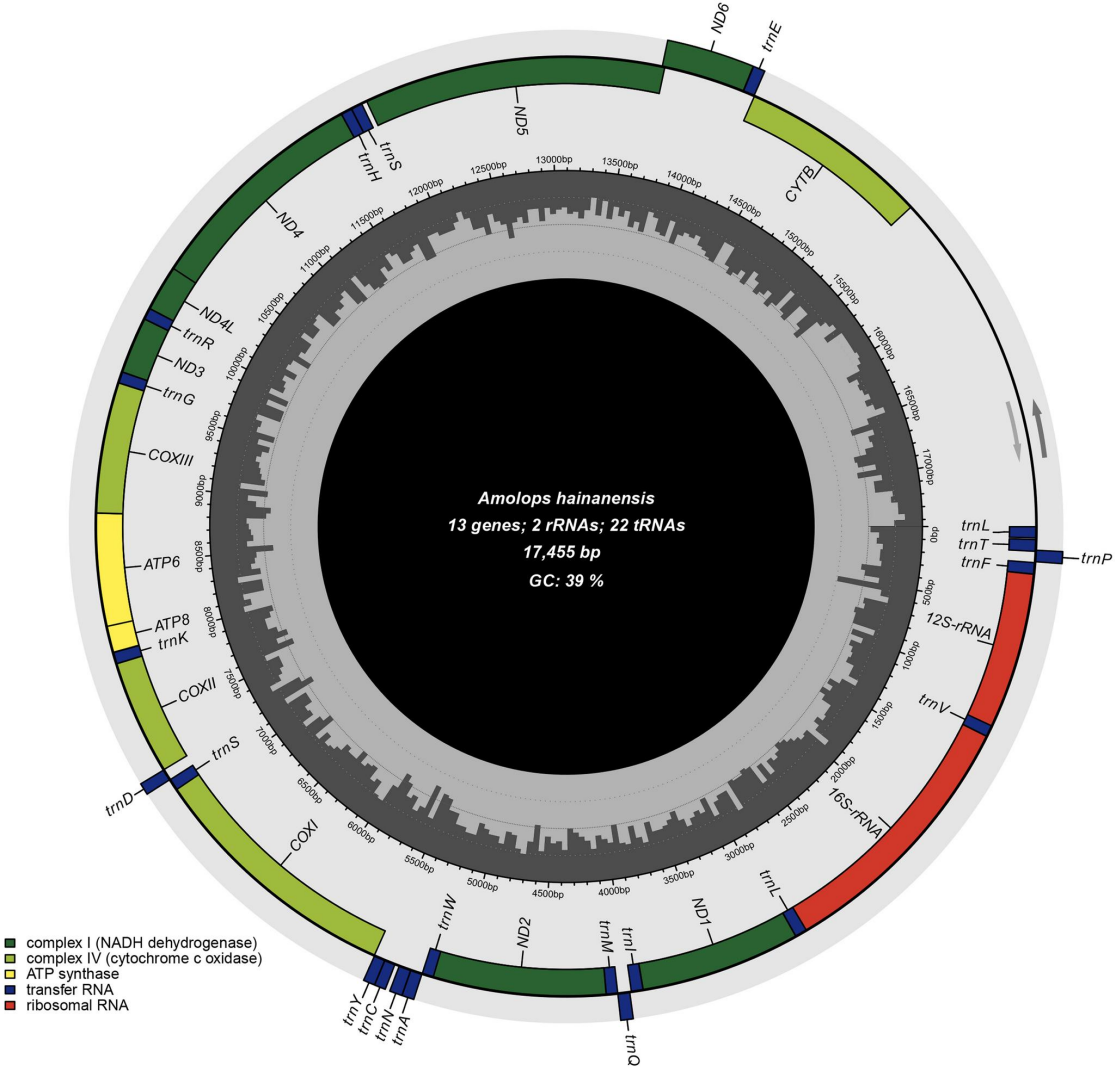
Mitochondrial genome map of *A. hainanensis*. Arrows indicate the direction of translation of genes. GC and AT contents across the mitochondrial genome are shown with dark and light shading, respectively, inside the inner circle.

The circular mitochondrial genome of *A. hainanensis* was successfully assembled (PP734094), with a length of 17,455 bp and an average coverage of 307 (Figure S1). The mitogenome of *A. hainanensis* is composed of 13 PCGs, 22 tRNA genes, 2 rRNA genes and one control region (Figure 3). Twelve PCGs initiate with the ATD codon, and one PCG (*COXI*) initiates with the GTG codon. Most of the termination codons in PCGs are predicted to be complete TAAs or incomplete codons T(aa), whereas *COXI* and *ND5* contain the codons AGG and *ND6* contain a codon AGA.

The nucleotide composition of this mitogenome is A 29.62%, C 24.54%, G 14.11%, and T 31.73%, with an AT-biased content of 61.36%. The mitogenomic phylogenies of 11 *Amolops* species were successfully reconstructed, with strong support assessed by both Bayesian posterior probability (PP) and ML bootstrap proportions (BP) (Figure 2). All nodes within the phylogenetic tree had a PP of 1.00. Phylogenetic analyses have divided *Amolops* into two main clades. *A. hainanensis* is a sister to the subclade consisting of *A. hongkongensis*, *A. sinensis*, *A. wuyiensis* and *A. ricketti*.

## Discussion and conclusions

Mitochondrial rearrangement has diverse patterns in amphibians (Zhang et al. 2021). Many species of amphibians have been reported to have unique gene orders in their mitochondrial genomes, and even higher higher-order taxa (Zhang et al. 2021). Neobatrachia is a group of amphibians, all of which exhibit mitochondrial gene arrangements that differ from those typical of vertebrates (Irisarri et al. 2012). Like the mitochondrial genomes of most Neobatrachia species, the *trnL*, *trnT*, and *trnP* genes in *A. hainanensis* are arranged between the putative control region and *trnF*, which differs from the typical vertebrate arrangement (Irisarri et al. 2012; Zhang et al. 2021). The mitogenomic gene arrangement order of *A. hainanensis* is identical to that of all the other published mitogenomes of *Amolops* species (Huang et al. 2016; Li et al. 2014; Shan et al. 2016; Xue et al. 2016; Yuan et al. 2016; Zhang et al. 2016; Huang et al. 2019; Wang et al. 2021), which implies evolutionary conservation of mitochondrial genome organization in the genus *Amolops*.

In agreement with the findings of Zeng et al. (2020), the mitogenomic phylogeny revealed two major clades within the Chinese *Amolops* species (Figure 2). Similarly, the east clade was composed of three subclades, and *A. hainanensis* alone was a subclade sister to the clade consisting of other *Amolops* species of the east clade. All internal nodes within the phylogenetic tree presented strong branch support values, which indicates that the complete mitogenome could be a useful tool for investigating the phylogeny of *Amolops* and possibly even Ranidae.

## Supplementary Material

sm.docx

## Data Availability

The genome sequence data that support the findings of this study are openly available in the NCBI GenBank at https://www.ncbi.nlm.nih.gov/under accession no. PP734094. The associated BioProject, SRA, and BioSample numbers are PRJNA1177585, SRR31111552, and SAMN44443522, respectively.
