# Supplementary material for: The complete mitochondrial genome of Amolops hainanensis (anura: ranidae)
Source: Mitochondrial DNA B Resour. 2025 Mar 7;10(4):272–7. doi: 10.1080/23802359.2025.2475826 (PMC11892061; doi:10.1080/23802359.2025.2475826)
Supplement: sm.docx [file TMDN_A_2475826_SM8574.docx]

**Title:** **The complete mitochondrial genome of *Amolops hainanensis* (Anura: Ranidae)**

Chuhan Zi^1^, Zhengyan Zhou^1^, Lanying Xu^1^, Sufan Yu^1^, Lin Ding^1^, Ziyi Liu^1^, Longming Fu^1^, Lin Feng^1^, Xiuzhong Li^2^, Yu Zhou^3*^

^1^College of Life Science and Bioengineering, Shenyang University, Shenyang, China

^2^School of Chemical Safety, North China Institute of Science and Technology, Hebei, 065201, China

^3^College of Life Science, Shenyang Normal University, Shenyang, China

^*^Corresponding authors: zhouyu1988@outlook.com (Yu Zhou); [lixiuzhong@ncist.edu.cn](mailto:lixiuzhong@ncist.edu.cn) (Xiuzhong Li)


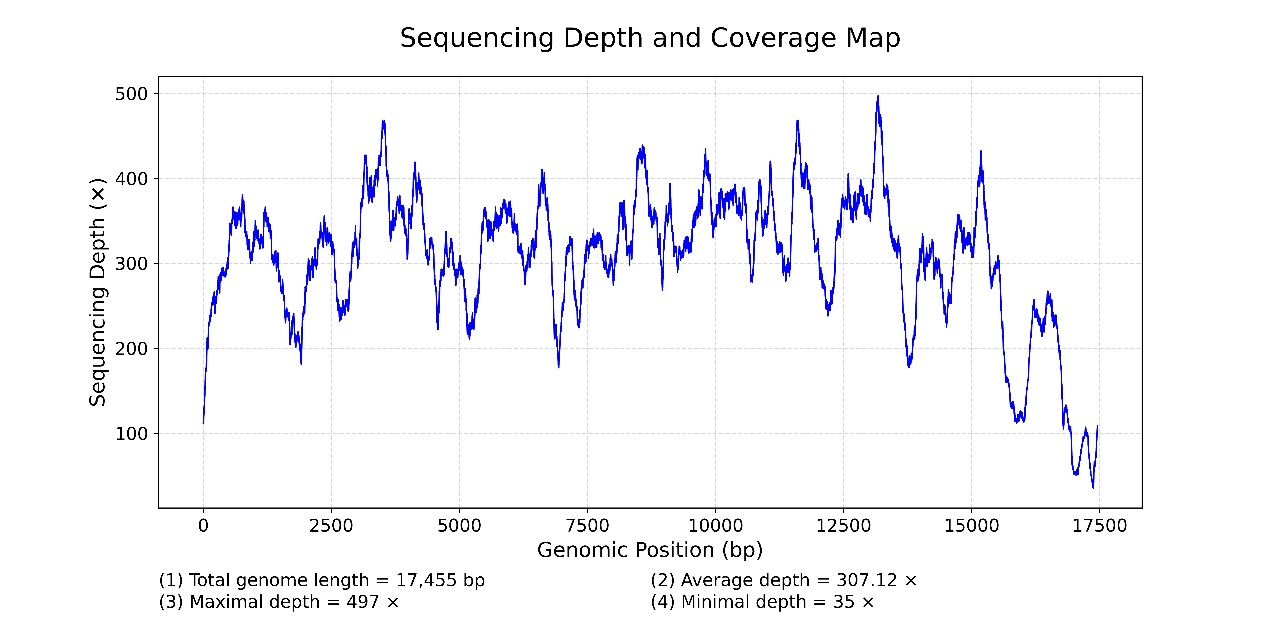


Figure S1. Sequencing depth and coverage map of the *Amolops hainanensis* mitochondrial genome.
